# Supplementary material for: Transcriptomics reveals the effects of NTRK1 on endoplasmic reticulum stress response-associated genes in human neuronal cell lines
Source: PeerJ. 2023 Apr 12;11:e15219. doi: 10.7717/peerj.15219 (PMC10105561; doi:10.7717/peerj.15219)
Supplement: Supplemental Information 1 [file peerj-11-15219-s001.docx]

**Supplemental Table S1**. Sequences of primers used for qRT-PCR analysis.

| **Gene symbol** | **Forward primer** | **Reverse primer** |
| --- | --- | --- |
| COL3A1 | GCCAAATATGTGTCTGTGACTCA | GGGCGAGTAGGAGCAGTTG |
| HSPA5 | CATCACGCCGTCCTATGTCG | CGTCAAAGACCGTGTTCTCG |
| XBP1 | CCCTCCAGAACATCTCCCCAT | ACATGACTGGGTCCAAGTTGT |
| COL1A1 | GAGGGCCAAGACGAAGACATC | CAGATCACGTCATCGCACAAC |
| CCND1 | CAATGACCCCGCACGATTTC | CATGGAGGGCGGATTGGAA |
| P4HB | GGCTATCCCACCATCAAGTTC | TCACGATGTCATCAGCCTCTC |
| THBS1 | TGCTATCACAACGGAGTTCAGT | GCAGGACACCTTTTTGCAGATG |
| GAPDH | GGAGCGAGATCCCTCCAAAAT | GGCTGTTGTCATACTTCTCATGG |
