# Supplementary material for: Transcriptomics reveals the effects of NTRK1 on endoplasmic reticulum stress response-associated genes in human neuronal cell lines
Source: PeerJ. 2023 Apr 12;11:e15219. doi: 10.7717/peerj.15219 (PMC10105561; doi:10.7717/peerj.15219)
Supplement: Supplemental Information 2 [file peerj-11-15219-s002.docx]

**Supplemental Table S2**. Mapping of clean reads on the reference genome.

| **Sample** | **NC_1** | **NC_2** | **NC_3** | **NTRK1_1** | **NTRK1_2** | **NTRK1_3** |
| --- | --- | --- | --- | --- | --- | --- |
| Total reads | 63133274 | 79151698 | 74787008 | 66648328 | 65338276 | 65664478 |
| Total mapped | 6118856 | 76709167 | 72315592 | 64894624 | 63667389 | 64089609 |
| Total Uniquely mapped | 57507735 | 71819148 | 67498872 | 60824004 | 59649972 | 59636680 |
| Total Multiple mapped | 3680828 | 4890019 | 4816720 | 4070620 | 4017417 | 4452929 |
| Total Pairs | 31566637 | 39575849 | 37393504 | 33324164 | 32669138 | 32832239 |
| Total Uniquely Concordant Pairs | 28178183 | 35168584 | 33072956 | 29769042 | 29244580 | 29249899 |
| Splice reads | 32062130 | 40549858 | 38136257 | 35248387 | 35187126 | 34535217 |
| Nonsplice reads | 25445605 | 31269290 | 29362615 | 25575617 | 24462846 | 25101463 |
