# Supplementary material for: Transcriptomics reveals the effects of NTRK1 on endoplasmic reticulum stress response-associated genes in human neuronal cell lines
Source: PeerJ. 2023 Apr 12;11:e15219. doi: 10.7717/peerj.15219 (PMC10105561; doi:10.7717/peerj.15219)
Supplement: Supplemental Information 3 — a The number of expressed genes > 0 to the number of total genes in genome. b The number of expressed genes ≥ 1 to the number of expressed genes > 0. [file peerj-11-15219-s003.docx]

**Supplemental Table S3**. Gene expression in genome.

| **Sample** | **Total genes in genome** | **Expressed genes (>0) (%)^a^** | **Expressed genes (≥1) (%)^b^** |
| --- | --- | --- | --- |
| NC_1 | 60649 | 22916 (37.78%) | 10990 (47.96%) |
| NC_2 | 60649 | 23109 (38.10%) | 10651 (46.09%) |
| NC_3 | 60649 | 22711 (37.45%) | 10485 (46.17%) |
| NTRK1_1 | 60649 | 22412 (36.95%) | 10580 (47.21%) |
| NTRK1_2 | 60649 | 22154 (36.53%) | 10364 (46.78%) |
| NTRK1_3 | 60649 | 22165 (36.55%) | 10438 (47.09%) |

^a^ The number of expressed genes > 0 to the number of total genes in genome

^b^ The number of expressed genes ≥ 1 to the number of expressed genes > 0
