# Supplementary figures and images for: Transcriptomics reveals the effects of NTRK1 on endoplasmic reticulum stress response-associated genes in human neuronal cell lines
Source: PeerJ. 2023 Apr 12;11:e15219. doi: 10.7717/peerj.15219 (PMC10105561; doi:10.7717/peerj.15219)

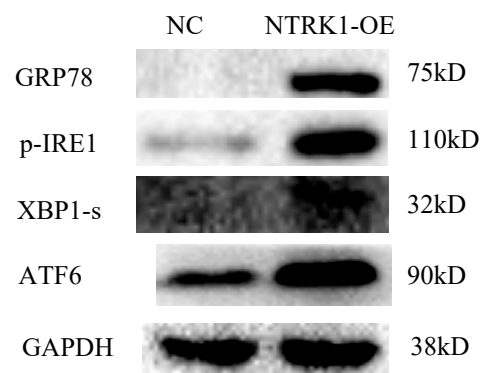

Supplement: Supplemental Information 5 [file peerj-11-15219-s005.pdf]

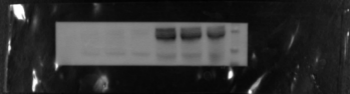

Supplement: Supplemental Information 8 [file peerj-11-15219-s008.pdf]

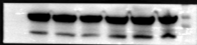

Supplement: Supplemental Information 9 [file peerj-11-15219-s009.pdf]
